# Mavacamten: Initial Insights From the Risk Evaluation and Mitigation Strategy Program

**DOI:** 10.1016/j.jacadv.2024.101430

**Published:** 2024-12-09

**Authors:** Matthew W. Martinez, Dewey Seto, Michael Cheung, Michele Coiro, Niki Patel, Arnaud Bastien, Jeffrey Lockman, Sonia Afsari, Milind Y. Desai

**Affiliations:** a Hypertrophic Cardiomyopathy Center, Morristown Medical Center, Atlantic Health System, Morristown, New Jersey, USA; b Bristol Myers Squibb, Princeton, New Jersey, USA; c Hypertrophic Cardiomyopathy Center, Heart Vascular Thoracic Institute, Cleveland Clinic, Cleveland, Ohio, USA

**Keywords:** hypertrophic cardiomyopathy, left ventricular ejection fraction, mavacamten, obstructive HCM, risk evaluation and mitigation strategy, septal reduction therapy

**Figure:**
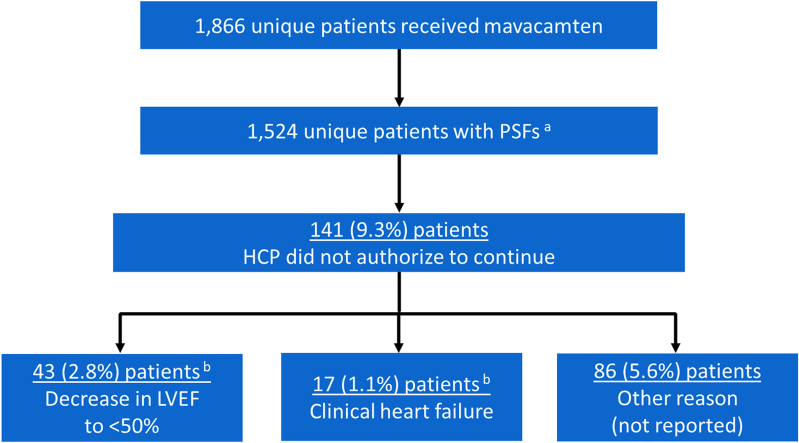

Hypertrophic cardiomyopathy (HCM) is a myocardial disorder characterized by pathological contractile function of the sarcomere leading to left ventricular (LV) hypertrophy, hyperdynamic contraction, and impaired diastolic function. The majority of these patients develop dynamic left ventricular outflow tract obstruction (obstructive HCM), which is associated with an increased risk of heart failure, arrhythmias, stroke, and sudden cardiac death.

Based on the phase 3 randomized clinical trial EXPLORER-HCM (NCT03470545), mavacamten (CAMZYOS) was approved by the U.S. Food and Drug Administration (FDA) in April 2022 for the treatment of adults with symptomatic New York Heart Association class II to III obstructive HCM to improve functional capacity and symptoms. Due to concerns related to the potential development of LV systolic dysfunction, clinical heart failure, and drug-drug interactions, mavacamten was approved under a risk evaluation and mitigation strategy (REMS) program.

We report here data required by the FDA as part of the REMS program for mavacamten during the first 10 months of real-world, post-approval use in the United States.

## Methods

Diagnosis and treatment decisions for patients with obstructive HCM were made by REMS-certified providers. Source data for the REMS program assessment report provided to the FDA are largely based on submitted forms completed by health care providers and pharmacies and are focused on compliance to 4 key program elements: 1) health care provider/designee/specialty pharmacist certification and training; 2) patient monitoring with echocardiograms; 3) screening for contraindicated concomitant medications; and 4) ensuring mavacamten dispensing occurs only when the previous 3 elements are completed and indicate it is safe to do so. Because the observational data presented here were required by the FDA as part of a REMS program, no ethics review was required. Statistical analysis was limited to reporting counts and percentages.

## Results

**Figure 1:**
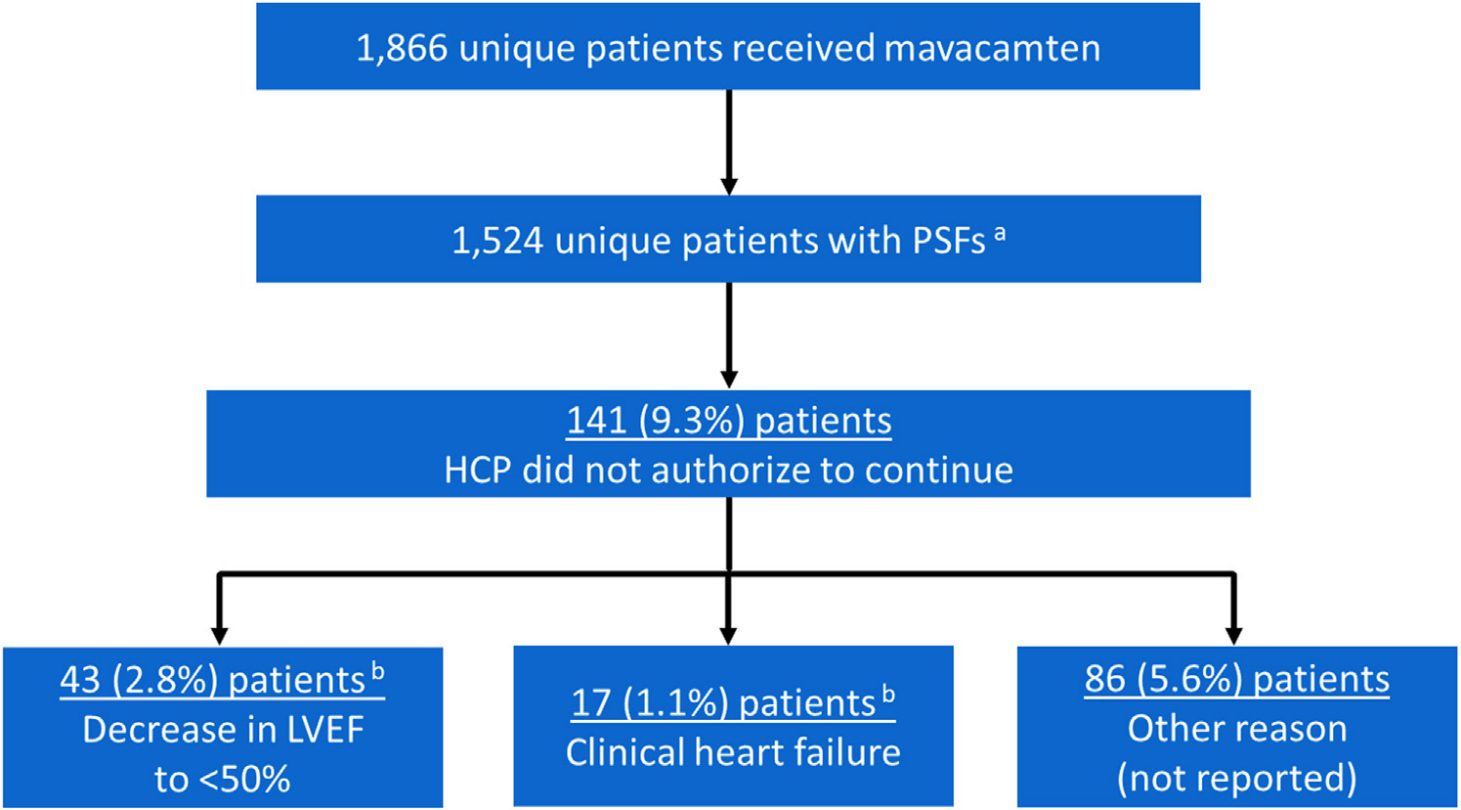
**Flow Diagram of Patients Who Received a Dispense of Mavacamten but Were Not Authorized to Continue Mavacamten Treatment** ^a^During the reporting period, 5,527 PSFs were expected. Of these, 5,094 (92.2%) were received and 433 (7.8%) were outstanding. ^b^5 patients reported both LVEF <50% AND a clinical heart failure event requiring hospitalization. HCP = health care provider; LVEF = left ventricular ejection fraction; PSF = patient status form.

Patients who received at least 1 dispense of mavacamten during the reporting period (N = 1,866) were ages 18 to 40 (n = 142, 7.6%), 41 to 60 (n = 571, 30.6%), and ≥61 years old (n = 1,153, 61.8%); 1,131 (60.6%) were women. Of the 1,524 unique patients with patient status forms submitted during the reporting period (Figure 1), 141 were not authorized by the provider to continue on mavacamten: 43 (2.8%) experienced a decrease in left ventricular ejection fraction (LVEF) to <50%; 17 patients (1.1%) experienced a clinical heart failure event requiring hospitalization (5 of these patients experienced a decrease in LVEF to <50% AND heart failure), and 86 (5.6%) patients were not authorized to continue for some other, unreported reason. Additionally, 8,057 Drug Interaction and Counseling Checklists were completed, though not all resulted in a dispense of mavacamten. Of these, 23 (0.3%) identified a concurrent contraindicated medication, which was discontinued in all cases prior to mavacamten dispense; 108 (1.3%) identified a concurrent medication that would require a mavacamten dosage reduction (57 resulted in a mavacamten dose reduction, 51 resulted, instead, in the discontinuation of the other medication). All identified potential drug interactions were resolved before mavacamten was dispensed.

Of the 7,454 authorized prescriptions dispensed during the reporting period, all (100.0%) were dispensed from certified specialty pharmacies, written by certified prescribers, dispensed to REMS-enrolled patients, were associated with completed patient status forms with documented echocardiograms, and were associated with completed Drug Interaction and Counseling Checklists with appropriate documentation.

## Discussion and conclusions

These data reflect important real-world clinical experience regarding mavacamten use, the largest publication of its kind. The 2.8% occurrence of LV dysfunction (defined as LVEF <50%) and 1.1% occurrence of clinical heart failure requiring hospitalization indicate the absence of significant LV dysfunction in this large group of patients and lack of a safety signal.

Previously published case series results have demonstrated favorable safety and efficacy of mavacamten in a real-world population.^1^^,^^2^ In addition, the recent 3.5-year open-label extension data further highlight the low incidence of a drop in LVEF (8.7% of patients) and clinical heart failure (6.1% of patients). Importantly, there appears to be no significant drop in LVEF from week 156 to week 180, a strong indicator of safety for patients who remain on therapy long term.*^3^* The present communication is consistent with those data which found an excellent safety profile of mavacamten with a low occurrence of safety events. The main limitations of those studies are that they were from single centers of excellence, and the number of patients taking mavacamten were low compared to other cardiac medications.

Given the excellent safety outcomes demonstrated by these data and the prior published case series studies illustrating minimal harm and high provider REMS compliance, one can conclude that the incidence of LV dysfunction associated with mavacamten treatment is consistent with previously published clinical trial data. It should be noted, however, that REMS data collection is designed to demonstrate that the required safe use conditions have been met and does not provide robust clinical data on safety, efficacy, or outcomes. Data capture using REMS does not provide exact values for LVEF (only whether or not LVEF decreased be below 50%) and does not allow us to determine if any adverse changes were caused by mavacamten or by comorbidities associated with HCM. Further data are needed to determine whether the frequency of echocardiograms specified by the REMS program could be decreased. Such a change would decrease the burden of the currently implemented schedule on patients and on the health care system.

## Funding Support and Author Disclosures

This work was funded in its entirety by Bristol Myers Squibb (BMS). Dr Martinez is an advisory board and steering committee member as well as an unbranded speaker for BMS. Mr Seto, Mr Cheung, and Ms Coiro and Drs Patel, Bastien, Lockman, and Afsari are employees of BMS. Dr Desai is a consultant for BMS, Cytokinetics, Viz.ai, Tenaya Therapeutics, and Edgewise Therapeutics.

## References

[1] Abdelfattah O.M., Lander B., Demarco K. (2023). Mavacamten short-term hemodynamic, functional, and electrocardiographic outcomes: initial real-world post-trial experience. JACC Adv.

[2] Desai M.Y., Hajj-Ali A., Rutkowski K. (2024). Real-world experience with mavacamten in obstructive hypertrophic cardiomyopathy: observations from a tertiary care center. Prog Cardiovasc Dis.

[3] Garcia-Pavia P., Oręziak A., Masri A. (Published online September 1, 2024). Long-term effect of mavacamten in obstructive hypertrophic cardiomyopathy. Eur Heart J.

